# Why Go to the Psychiatric Hospital? The Experiences of People Living with Mental Disorders Hospitalized Multiple Times in One Year: A Qualitative Study

**DOI:** 10.3390/nursrep15040132

**Published:** 2025-04-14

**Authors:** Patrick Martino, Michael Saraga, Jérôme Dubuis, Milja Kovacevic

**Affiliations:** Department of Psychiatry (DP), Centre Hospitalier Universitaire Vaudois (CHUV), 1008 Prilly, Switzerland; patrick.martino@chuv.ch (P.M.); jerome.dubuis@chuv.ch (J.D.)

**Keywords:** psychiatry, qualitative research, rehospitalizations, patient experience, patterns of care, hospital’s role, nursing

## Abstract

**Background**: Recent trends in public psychiatry have resulted in increasingly shorter hospital stays. In parallel, a number of patients seem to require frequent rehospitalization. Several studies have examined the subject of rehospitalization in psychiatry from a quantitative point of view, but little qualitative literature exists on patients’ experiences. **Objectives**: This qualitative study, conducted in a Swiss university hospital, explores the lived experiences of patients who are hospitalized multiple times within a year. **Methods**: Using criterion-based purposive sampling, we conducted 20 semi-structured interviews. **Results**: Our findings show that patients explain their return to the hospital either by external factors, such as social and housing difficulties, or by their state of suffering and the sense of security that the hospital can offer them. From their perspective, hospitalizations are not always failures but can also meet a specific need for safety and stabilization. **Conclusions**: This study highlights the importance of better understanding the needs of frequently hospitalized patients to optimize their care, while also emphasizing the value of exploring their lived experiences through qualitative research.

## 1. Introduction

The deinstitutionalization of psychiatry and advances in medical treatment have radically altered the way psychiatric patients are treated. Since the 1960s, psychiatric asylums have gradually given way to the hospitals we know today, designed to treat crises and rapidly reintegrate patients into society [1].

In Switzerland, between 1992 and 2011, the number of psychiatric beds fell by 44%, while the number of hospitalizations over the same period rose by 151% [2]. Between 2012 and 2022, the number of beds per capita slightly decreased (−5.4%) while the number of hospitalizations kept growing steadily (+6.7%) [3]. This paradigm shift, aimed at strengthening outpatient care and reducing hospitalization to a strict minimum, seems not to suit all patients. A significant proportion of hospital admissions is linked to patients who are hospitalized multiple times. This clinical observation has been confirmed by several studies of care trajectories [1,4,5].

A recent Swiss study [1] suggests that 15.8% of hospital days over a three-year period are attributable to just 2.9% of patients who very frequently attend the psychiatric hospital.

The phenomenon of patients going through a pattern of frequent hospitalizations in psychiatry is often seen as a problem in itself, both because it contributes to hospital congestion and because it tends to be perceived as a failure of previous hospitalizations. The effectiveness of inpatient and community care is commonly assessed through the rate of readmission within 28 days of discharge, which serves as a key quality indicator on an international scale [6,7,8]. Although the validity and practical relevance of using readmission rates in this manner remain a topic of debate [9], minimizing readmissions remains a priority for healthcare services aiming to enhance efficiency and improve patient outcomes. For these reasons there is interest in the literature in this population, which has mostly been described quantitatively [1,2,3,4,5,10], with overlapping results: people most at risk of frequent readmission are generally younger, single, with a low level of education, substance-related problems, and psychotic disorders, and are more often affected by somatic comorbidities.

Despite the extensive quantitative results, there are little qualitative data on the experiences of people who are frequently rehospitalized in psychiatry.

One qualitative study performed in Australia [11] that investigated the perceptions of frequently rehospitalized patients underlines the intricate nature of the readmissions phenomenon and explains them as the struggle of some people to survive in an apparently inhospitable world. Another study [12] carried out in six European countries also identified the theme of rehospitalization as a part of the complex recovery process for frequently hospitalized patients in psychiatry. Both of these studies suggest that frequent hospital readmission is not necessarily a simple statistic that should be eradicated, and that further research should be performed in the field of patients’ experiences of hospital readmissions.

As of now, no qualitative studies on this specific matter exist in Switzerland. This qualitative study aims to shed light on the understanding and experiences of psychiatric hospitalization among patients who are hospitalized multiple times in one year.

## 2. Materials and Methods

### 2.1. Study Design

This was a monocentric qualitative descriptive study conducted in a university hospital in French-speaking Switzerland. The research protocol was validated by the cantonal ethics commission in November 2023 (CER-VD 2023-01835).

This study design was employed to address the paucity of research on the experience of frequently hospitalized patients, as it offers the possibility of highlighting individuals’ lived experiences and perceptions in nursing research [13].

### 2.2. Recruitment

Study participants were selected by criterion-based purposive sampling from inpatients in a hospital providing acute psychiatric care for the adult population (18 to 65 years). The department has 90 beds divided among five units, with an occupancy rate of 99% and an average length of stay of 17.6 days [14].

Inclusion criteria for study participants were age (18–65 years), at least three hospitalizations (including the current one) in a psychiatric unit in Switzerland in the 12 months preceding the interview (regardless of the mode of admission), and a good level of spoken French.

Exclusion criteria were the presence of a diagnosis of moderate or more severe intellectual deficit and incapacity for discernment at the time of the interview. Patients unable to provide informed consent were not eligible to participate.

Patients eligible for the study were identified by criterion-based purposive sampling by consulting with the nursing teams and head nurses. Using administrative management software and electronic files, an initial check of eligibility criteria was carried out (age and number of hospitalizations). Formal verification of inclusion and exclusion criteria was then carried out once the patient’s informed consent had been obtained.

Eligible patients were met by the first author during an initial visit to present the nature of the study, its purpose, the procedures involved, the expected duration, the potential risks and benefits, and any discomfort it may entail. Each patient was informed that participation in the study was voluntary, that withdrawal from the study at any time was possible, and that withdrawal of consent would not affect future medical care and/or treatment. A signed informed consent form was collected for every participant in the study.

In qualitative descriptive research, sample sizes are usually small and difficult to pre-determine. Following qualitative descriptive guidelines [13], data saturation was used as a sample size criterion and was reached after 20 interviews, at which point no new information emerged from the data. A total of 39 people were approached during the recruitment process, 19 of whom declined to participate. Refusal to participate was often motivated by a lack of interest in the research topic or by the need to be recorded during the interviews.

### 2.3. Data Collection

Participants (n = 20) (Table 1) were invited to take part in a semi-structured interview based on an interview guide (Appendix A).

The average age of the 20 participants (12 men and 8 women) was 36.9 years. Fourteen had been admitted voluntarily (70%) and six through forced hospitalizations (30%). The average number of hospital admissions over the previous 12 months for all participants was 3.7. In total, 40% of patients presented a psychotic disorder as their main diagnosis, 35% a mood disorder, and 25% a personality disorder. Interviews lasted between 25 min and 75 min, with an average of 49 min. All participants had a good level of spoken French.

The interviews were carried out in a meeting room in the hospital and were conducted by the first author, who is a nurse with 6 years of clinical experience and a specialization in clinical research. The interviews were recorded using a portable audio recorder. The first author was supervised in this phase by the clinical nurse specialist and the senior physician, both of whom have significant experience in qualitative research and in conducting qualitative interviews.

The interview guide (Appendix A) was designed as a funnel, allowing patients to express themselves freely about their experience and understanding of the reasons for hospitalization. The time devoted to this first part varied from patient to patient. When the interviewee provided no further information in response to the open-ended questions, the interview then turned to the more specific issues arising from quantitative studies and associated with repeat rehospitalizations.

### 2.4. Analysis

The interviews were transcribed in full, in verbatim form, using Sonix software (https://sonix.ai/, accessed on 28 February 2025) [15]. Each verbatim interview was then reread by the interviewer while listening to the audio recording to ensure the accuracy of every text. Thematic analysis was conducted using a data-driven approach, following the phases described by Braun and Clarke [16]: familiarization with the body of data, code generation, theme identification, theme review, theme definition and nomination, and finally report writing.

The research team was made up of a clinical research nurse (first author), a clinical nurse specialist, a senior physician, and one nursing manager. Familiarization with the body of data and coding was carried out using MAXQDA Analytics Pro 2022 [17]. Every transcript was read several times by the clinical research nurse and the clinical nurse specialist to generate the initial codes and groups of codes, from which underlying themes eventually emerged. To ensure methodological rigor in coding and codebook development, seven interviews were coded in parallel by the principal investigator and the clinical nurse specialist. The codes were discussed together, and once a consensus had been reached, all data were coded according to the revised codebook. The identification, description, naming, and review of the themes was performed iteratively with the whole research team by continuously reviewing the data until a consensus was reached. During this phase a certain level of interpretation was inevitable, but efforts were made to keep the themes as close as possible to the participants’ experiences and narrative.

## 3. Results

**Figure 1:**
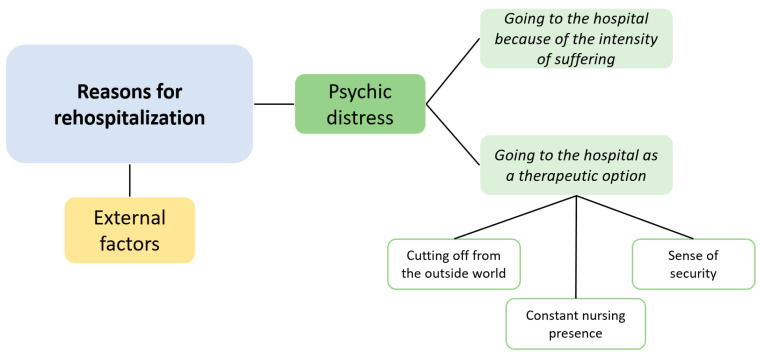
Summary of thematization.

The patients’ stories spoke richly of the factors that contributed to their psychiatric hospitalization (Figure 1).

Understanding the reasons for repeated rehospitalization is complex. Patients often described paths that lead to hospitalization as mixed and intertwined. This complexity was well described by one patient:

Pers. 16: *“It’s a whole. There’s a whole that made it spill over. It’s not, we can’t target one element, it’s several elements that have interlocked to form a big ball of wool. And then we can talk about the big ball of wool. You can say that there are several threads of different colors, but it’s still a big ball of wool…”*.

A first thematization was related to the explanations the participants gave for their hospitalization, which fell into two broad categories, which we named “psychic distress” and “external factors”. “Psychic distress” as a reason for hospitalization implies a therapeutic role for the hospital, defined in a positive or negative (“no other option”) way. “External factors” explain the hospitalization as resulting from a problematic environment or insufficient, incompetent outpatient care.

**Psychic distress:**

Participants often explained their return to the hospital as being because of a state of suffering, more or less explicitly related to the presence of a mental illness. They talked of “recurring malaise”, “worsening of symptoms”, “being exhausted”, or any other form of exacerbation of their suffering that brought them to the hospital. This theme can be split in the following sub-themes.

**Going to the hospital because of the intensity of suffering:**

Patients explained their arrival at the hospital due to intense psychological distress. Some patients described their admission as a somewhat passive experience, independent of voluntary or forced admission, where the hospitalization was seen as unavoidable because of the absence of other solutions. The interviewed patients presented different levels of insight: some struggled to put precise words to their suffering and spoke of a feeling of unease without precision that led them to the hospital. They often talked about being overwhelmed and fed up, of an accumulation of suffering and discomfort that made the hospital the only option. However, the patients in question were unable to precisely identify the symptoms of their illness or to qualify the extent of their discomfort. One patient said:

Pers. 14: *“Well, here I come. Because I can’t… I can’t manage my life anymore…”.*

Another patient, voluntary admitted to the hospital, explained:

Pers. 6: *“Yes, I chose to come here. I feel I’m not well and I come here”.*

Other patients could identify quite precisely the nature of the psychological suffering that brought them to the hospital. For example, one woman very clearly explained the deterioration in her psychological state before hospitalization:

Pers. 10: *“It’s mostly because of dark thoughts, psychotic symptoms that are pretty constricting we’ll say, visual hallucinations. Maybe I had an auditory hallucination at the beginning of my hospitalization, but I’m not sure. There were psychotic symptoms. Lack of motivation. I found it hard to do everyday things. I found it very difficult to be alone. I was often accompanied by friends or family. And then we were all exhausted and I myself was totally exhausted”.*

**Going to the hospital as a therapeutic option:**

Some interviewed patients clearly identified reasons why hospitalization is beneficial, adding an active dimension to the act of going to the hospital regardless of the admission mode. Hospital admission was often perceived as a source of relief or respite from overwhelming responsibilities and seemingly insurmountable challenges outside. The hospital environment was generally seen as comfortable, providing essential needs like food and shelter. They expressed feeling cared for and appreciating the opportunity to receive support. The interviewees talked about specific therapeutic roles of the hospital, as detailed later. Some findings suggest that the participants did not come to the hospital only in the absence of other solutions, as they believed that the hospital also has therapeutic value:

Pers. 12: *“If I am here and I come, it is for a certain reason. It is to strengthen myself”.*

For another person, the hospital was complementary to outpatient care, a genuine therapeutic option among others:

Pers. 10: *“For me, [the hospital is] really an integral part of my care. It really complements outpatient care in times of crisis when I can’t be on my own, when I need assistance every day”.*

The following sub-themes outline more precisely what is beneficial about coming to the hospital for frequently hospitalized patients.

**Sense of security:**

Some patients described the hospital as a safe place, a bubble, a secure environment providing a sense of security:

Pers. 8: *“If I don’t feel safe, it’s because the work hasn’t been done. So that’s why I come back to the hospital, where I can be in a safe environment. But at the same time I can work on my problems”.*

Another patient stated:

Pers. 9: *“[The hospital] is like a bubble in which I say to myself, okay, here I am, in a room that’s empty, that’s tidy, so I don’t have all my stuff and everything. So here I can start from scratch”.*

**Constant nursing presence:**

A sub-theme also identified was the constant presence of health care professionals, especially nurses, who are available day and night. Participants often contrasted this to outpatient care, where availability is more limited, which sometimes proves insufficient for these patients:

Pers. 10: *“It’s at times like these that I come to the hospital, it’s really when I need to communicate every day with nurses and professionals. And then I need to be supported a little more closely than just in outpatient care”.*

**Cutting off from the outside world:**

Often identified was also the need to be cut off from the outside world. Participants described the hospital as a cocoon that allows them to escape from their difficulties. The stressors of their life are put on hold, offering an opportunity to refocus on themselves:

Pers. 10: *“There’s also the aspect of being cut off from real life. You’re in a bubble, you’re in a place where you rest, where you get back to basics, where you try to take care of yourself, something you didn’t necessarily have time for outside because there are responsibilities and all that. So it’s as if we’re forced to concentrate on ourselves and nothing else. So there’s no need to cook, no need to clean. Mundane things like that, daily mundane things like that, which can become a constraint, which can become a burden depending on how we feel”.*

**External factors:**

As stated above, external factors included outpatient care that was perceived as insufficient or incompetent, as well as social problems, notably related to finances or housing. Medication is an important feature of the first category. Participants said, for example, that they return to hospital because their medication was not adapted to their situations:

Pers. 3: *“I tell myself that if I’d had another antidepressant, I might not have ended up here [in the hospital]”.*

Another patient also described the deterioration of her condition as being because of inappropriate medication, which led her to deviate from the suggestions of the healthcare professionals and to self-medicate:

Pers. 4: *“Because the treatments I was given [before the hospitalization] weren’t effective, so I was self-medicating”.*

Examples of housing difficulties leading to hospitalization included homelessness:

Pers. 8: *“[Coming back to the hospital] is also possible because the fact of being, of wondering every day where one’s going to sleep, how one’s going to do this, that can have an impact on morale”.*

Other difficulties can be ascribed to an environment that is not sufficiently responsive to the patient’s needs:

Pers. 13: *“I’m in an assisted living facility. And then the team, there are maybe two or three of them a day, so for me it’s not a supportive enough environment”.*

## 4. Discussion

The analysis of the 20 patients’ interviews enabled us to gain a better understanding of the complex health and social issues involved in psychiatric hospitalizations for frequently hospitalized patients.

The attribution of hospitalizations to external factors is consistent with the quantitative literature on the subject. Patients largely refer to known factors such as medication adaptation, the unsuitability of their living environment, and inadequate outpatient care [1,2,18].

The participants’ perspectives on the hospital also say something about its perceived role in their care trajectories. Contrary to the common view of rehospitalization as a failure of the healthcare system [19], some patients’ accounts suggest that repeated stays in the hospital may be a stage in their care pathway, a useful complement to outpatient care. In this view, there is a dynamic and iterative articulation between the hospital setting and other actors in the healthcare system.

This more positive perception of the hospital contrasts with historical representations of psychiatric establishments, often described as closed and coercive places [20,21,22]. Participants in this study tended to recognize the hospital as a place of care and recovery, a cocoon, a safety net, and a therapeutic environment.

There are limited qualitative studies on readmission, but a study in Australia [11] found that readmission is a complex and ongoing process rather than a discrete event. The Australian study shows that patients often use coping strategies when they are distressed. If these strategies fail, patients may choose to go to hospital, or it may be imposed on them. For many participants, as our findings also suggest, admission serves as a refuge, offering temporary relief, stability, and an opportunity to regroup, despite its inherent restrictions. Rather than attributing readmission solely to individual pathology or systemic service failures, the Australian study’s findings overlap with ours and emphasize the crucial role of environmental and social factors in perpetuating the readmission cycle. In this context, a more comprehensive response seems necessary, integrating health, housing, and employment support services to create sustainable conditions for community living.

A different study conducted in multiple European countries [12] also backs the idea that being readmitted to a facility is not a one-time occurrence, but rather a continuous journey influenced by both personal and systemic elements. Similarly to our findings, several patients see rehospitalization as a form of comfort and safety, an escape from pressures, and a way to restore balance. Nonetheless, this situation is often likened to the “tip of the iceberg,“ hinting at issues that still linger, unresolved, in society. In the shift towards a recovery-focused method in health care services, it is increasingly acknowledged that effective treatment should go beyond mere hospital stays and encompass comprehensive support networks.

The constant presence of healthcare professionals was also highlighted in these studies [11,12] and in our findings, more specifically the presence of nurses, with whom the therapeutic relationship is highly valued. Multiple qualitative studies [23,24] have addressed the importance of the therapeutic relationship with caregivers in the psychiatric hospital setting. A positive therapeutic relationship contributes to a feeling of security and mutual respect and to a more positive perception of hospitalization. In this context, nurses play a crucial role, as they are the most consistently present professionals throughout the day.

These accounts seem to suggest that, for some patients, the cycle of rehospitalization is a necessary, well-perceived part of their care pathway, while for others it is an unwanted passage explained by their intense psychic suffering.

This contrast raises the question of the need to eradicate the phenomenon of rehospitalization and of the use of statistics on readmission rates to the hospital as a reliable indicator of the quality of care that can be offered [9]. This complexity means that patients’ opinions and experiences must be taken in great account, in order to better understand the issues at stake when they leave the hospital. In this context, the importance of transitional care has been advocated: transitional care enables better connection with the outpatient network and increases patients’ empowerment in healthcare decision-making [25,26].

There are a number of limitations to this study that need to be highlighted. First, the qualitative nature of the approach, while rich in depth and nuance, restricts the generalizability of the results to the entire population concerned. Second, the choice of participants may have introduced selection bias, as the stories collected were potentially influenced by the patients’ availability, their willingness to participate, or their personal experience of care. This bias may account for the generally positive perception of the phenomenon of frequent rehospitalization. While this subjectivity is at the heart of the richness of qualitative data, it requires careful interpretation to avoid over-generalization. Finally, the results from this study were obtained in a specific institutional and geographical context, which may limit their transferability to other psychiatric care environments.

## 5. Conclusions

The role of the psychiatric hospital has evolved over recent decades. Despite deinstitutionalization efforts, many patients still need the hospital setting to progress in their recovery process. Even though many hospital admissions remain unwanted, most participants identified the hospital as a response to a need that cannot be met by another provider or service. This observation contrasts with the historical image of psychiatric hospitals and also provides a more positive view of frequently rehospitalized patients. Readmission is not simply a problem to be solved; rather, it can be seen as a process that is potentially beneficial to some patients. Thus, readmission rates are not necessarily a precise indicator of the quality of the care that is provided in a hospital setting.

This study underlines the complexity of psychiatric hospitalization dynamics, which are part and parcel of patient care and illness trajectories. By examining this phenomenon from the point of view of the people concerned, we have identified difficulties known to exist in the system, but also new keys to understanding the positive features of hospital care. Our findings, as well as the limitations of our study, underline the importance of patients’ perspectives in exploring a complex healthcare phenomenon and pave the way for future research into understanding repeated hospitalizations and the role of today’s psychiatric hospital.

## Figures and Tables

**Table 1:** Socio-demographic table.

ID	Age	Sex	Admission	Main Diagnosis	Number of Hospitalizations During the 12 Months Before the Interview
Pers. 1	64	F	Forced	Psychotic disorder	7
Pers. 2	24	M	Forced	Psychotic disorder	4
Pers. 3	24	M	Voluntary	Personality disorder	3
Pers. 4	22	F	Voluntary	Mood disorder	3
Pers. 5	32	M	Voluntary	Psychotic disorder	3
Pers. 6	42	M	Voluntary	Psychotic disorder	4
Pers. 7	24	M	Forced	Psychotic disorder	3
Pers. 8	27	M	Voluntary	Personality disorder	3
Pers. 9	25	M	Voluntary	Personality disorder	3
Pers. 10	28	F	Voluntary	Mood disorder	3
Pers. 11	55	F	Forced	Mood disorder	3
Pers. 12	29	M	Voluntary	Psychotic disorder	3
Pers. 13	26	F	Voluntary	Personality disorder	8
Pers. 14	55	M	Voluntary	Personality disorder	5
Pers. 15	56	F	Forced	Mood disorder	3
Pers. 16	37	M	Forced	Psychotic disorder	3
Pers. 17	21	M	Voluntary	Psychotic disorder	3
Pers. 18	38	M	Voluntary	Mood disorder	3
Pers. 19	61	F	Voluntary	Mood disorder	3
Pers. 20	47	F	Voluntary	Mood disorder	4

## Data Availability

The data that support the findings of this study are available from the corresponding authors upon reasonable request.

## Public Involvement Statement

No public involvement in any aspect of this research.

## Guidelines and Standards Statement

This manuscript was written according to Consolidated Criteria for Reporting Qualitative Research (COREQ) guidelines.

## Appendix A. Interview Guide

**Themes**	**Question**
Generic understanding of readmissions (15′)	- What brings you to the hospital today? - You have already been hospitalized this year. How do you explain this return? - What would you have changed upon discharge from your last hospitalization to avoid this readmission?
Ambulatory care (medical, nursing, case manager, etc.) (3′)	- How was outpatient follow-up organized when you were discharged? - What worked well during this outpatient follow-up? - What could have been improved about your outpatient care? - What impact did your outpatient treatment have on your return to hospital?
Medication (psychotropic treatment) (3′)	- After your discharge, did you continue your prescribed treatment? - What were the advantages of this treatment? - What could have been improved about your medication? - What impact did your medication have on your return to hospital?
Accommodation (apartment, hotel, hostel, etc.) (3′)	- How would you describe your housing situation at discharge? - What were the positive aspects of your housing situation? - What could have been improved about your housing situation? - What impact did your housing situation have on your return to hospital?
Financial situation (income, pensions, etc.) (3′)	- How would you describe your financial situation at discharge? - What was positive about your financial situation? - What could have been improved about your financial situation? - What impact did your financial situation have on your return to hospital?
Use of psychoactive substances (alcohol, cannabis, cocaine, heroin, etc.) (3′)	- Do you use psychotropic substances? - What impact––if any––did this consumption have on your readmission to hospital?
Final considerations (10′)	- Is there anything else you would like to add about the reasons for your readmission to hospital?
